# Predicting Youth Justice supervision among young people placed in out-of-home care before age 10.

**DOI:** 10.23889/ijpds.v7i3.1971

**Published:** 2022-08-25

**Authors:** Catia Malvaso, Pedro Santiago, Rhiannon Pilkington, Alicia Montgomerie, John Lynch

**Affiliations:** 1 University of Adelaide

## Objectives

To investigate how well we can predict which young people will be under Youth Justice (YJ) supervision by age 18, among children placed in out-of-home care (OOHC) before age 10.

## Approach

Data were drawn from the Better Evidence Better Outcomes Linked Data (BEBOLD) platform, which includes whole-of-population linked administrative data on ~500,000 children in South Australia born 1991 onwards. In this study, children born 1991-1998 were followed from birth to age 18. Logistic regression models were used to predict the probability of a child placed in OOHC before age 10 transitioning into YJ by age 18. YJ contact was defined as at least one community- or custodial-based supervision order. Child and maternal sociodemographic and perinatal characteristics at birth, as well as maltreatment and placement characteristics were included as predictors.

## Results

Collaboration has been cyclical, with results from the qualitative research and discussion with the reference groups informing the initial quantitative research direction. Findings from this research were presented back to the groups, resulting in further questions and directions to explore.

The journey so far has been one of learning and understanding the skills I have and the role they can play whilst acknowledging the limits of my own knowledge and the need for Indigenous voices to guide the research in order to be doing research with Indigenous peoples, rather than on them.

## Conclusion

A total of 2,832 children experienced OOHC before age 10. Of these 13.5% (n=381) experienced contact with YJ by age 18. Model discrimination (AUROC) was 0.8. Using the top 30% of the predicted probabilities as the ‘high’ risk threshold: 523 children were classified as ‘high’ risk; sensitivity was 69.8%; specificity was 76.5%, and the positive predictive value was 32.3%. The prediction model improved classification of those children who go on to experience YJ supervision from 13.5% of all of the 2,832 eligible children, to 32.3% of those in the highest 30% of risk.

